# Effects of avoidance in German military police resilience training

**DOI:** 10.1080/08995605.2025.2525655

**Published:** 2025-07-05

**Authors:** Ansgar Johannes Dietmar Herchenröder, Robert-Jacek Gorzka, Philipp Yorck Herzberg, Niels Brinkmann

**Affiliations:** a Faculty of Humanities and Social Sciences, Helmut-Schmidt-University/University of the Federal Armed Forces Hamburg, Hamburg, Germany; b Department of Military and Operational Psychology, Bundeswehr Military Police Command, Hanover, Germany; c Personality Psychology and Psychological Assessment, Helmut-Schmidt-University/University of the Federal Armed Forces Hamburg, Hamburg, Germany

**Keywords:** Avoidance, military police, coping, resilience training, qualitative content analysis

## Abstract

Resilience is a vital psychological resource for military police personnel, who routinely operate in high-stress, high-risk environments that demand rapid decision-making, emotional regulation, and sustained mental stamina. While resilience training is increasingly integrated into military structures to foster adaptive coping and psychological endurance, the role of the psychological phenomenon avoidance – a commonly used but often maladaptive coping strategy – remains insufficiently examined in this context. This qualitative study explores how avoidance is experienced and managed among German military police personnel and how it may impede the development of resilience. Guided by two central research questions—(1) How does avoidance manifest itself? and (2) How do military police personnel deal with avoidance? – nineteen semi-structured interviews were conducted with active-duty military police members. Data were analyzed using qualitative content analysis. Findings reveal that avoidance is commonly perceived as the evasion of distressing experiences and is employed across a range of scenarios, both in routine duties and high-pressure situations. Avoidance emerges on cognitive, emotional, and behavioral levels, indicating a complex, multi-dimensional pattern. While some interviewees displayed growing awareness of their avoidance tendencies and a willingness to confront them, others remained ambivalent or unaware of their impact. Importantly, participants voiced a clear need for structured support, specifically through resilience-building training tailored to address and reduce avoidance behaviors. These findings suggest that the inclusion of targeted strategies for recognizing and mitigating avoidance should be a priority in future resilience training programs. The study offers implications for designing psychological support within military contexts and highlights key areas for further research.

**What is the public significance of this article?—**This study found that military police officers often use psychological avoidance to cope with stress, which can harm performance and mental health. Officers expressed a need for resilience training to address these challenges. Tailored programs can help them face stress more effectively, improve well-being, and enhance operational readiness, emphasizing the importance of investing in targeted mental health and training initiatives for military personnel.

Bundeswehr military police personnel face distinctive and persistent challenges both at home and abroad. These include unclear operational contexts, extended absences from home, and specialized roles like personal protection or facility security (von Krause, 2017). Such recurring stressors can impair mental health, physical well-being, and performance. To counter these effects, resilience is considered essential.

Depending on the context and timing, individuals with greater resilience can access more resources – or use them more effectively – to manage high-stress situations. This is reflected in the quality of their responses and the outcomes of the coping process (Arnold et al., 2023). Hence, providing them with appropriate coping strategies to foster individual resilience against these stressors is paramount (Adler et al., 2015; Angehrn et al., 2023; R. J. Gorzka & Lorei, 2024).

Based on these theoretical foundations, training programs have been developed to enhance resilience among military police personnel and similar executive forces, translating theory into practice (Adler et al., 2009; Andersen et al., 2015; Crane, Boga, et al., 2019; Doody et al., 2021; Kho et al., 2022; Niederhauser et al., 2023; R. J. Gorzka et al., 2023).Resilience training is based on the assumption that resilience is a variable capacity that can be strengthened by building appropriate competencies and resources (Masten, 2014), with protective factors enabling a reduction in the negative consequences of stress (Fletcher & Sarkar, 2013). These usually include coping skills for managing stress and self-reflection in the sense of adaptively handling stressors and concomitant symptoms (e.g., Adler et al., 2015; Andersen et al., 2015; Angehrn et al., 2023; Crane et al., 2018; Foran et al., 2012; Forbes & Fikretoglu, 2018). The aim is not only to provide participants with techniques for adaptively coping with stress, but also to encourage them to deal independently and effectively with their shortcomings and thus initiate their personal development (*growth*, e.g., Yeager and Dweck, 2012), which should lead to better health, satisfaction, and increased performance in the long term. On this assumption, engaging properly with their personal deficits ultimately promotes resilience (R.-J. Gorzka et al., 2022).

Military police personnel’s resilience largely depends on actively confronting the challenges they face in training and deployment. This process can be uncomfortable, especially when increased self-awareness reveals previously repressed conflicts between external demands, personal expectations, and actual performance levels (Greif, 2008). Recognizing one’s weaknesses, mistakes, or negative traits can create tension, as it conflicts with the natural tendency to protect one’s self-image (*defense mechanism*; Silverman & Aafjes van Doorn, 2023). For example, some individuals avoid social situations due to fear of negative evaluation, which can lead to isolation and loneliness, restrict positive social experiences, and reinforce the belief of being socially inadequate (Trew, 2011). Ultimately, such behavior may weaken unit cohesion when it comes to military training or deployment.

In such instances, individuals often seek to mitigate discomfort by either preventing the situation from occurring or withdrawing from it once it has arisen (Steingräber et al., 2023). This behavior is referred to in the present article as *avoidance*. More broadly, Hayes et al. (1996) conceptualized this phenomenon as *experiential avoidance*, describing the tendency to evade situations or actions anticipated to cause distress or pose a perceived threat (e.g., Servatius, 2016; Wang et al., 2024). Within both clinical and operational psychology, the term is frequently employed to characterize individuals with affective or trauma-related disorders who avoid triggers that could elicit the reexperiencing of traumatic events (Bisson & Olff, 2021). However, in the present context, avoidance is considered a common, everyday coping strategy that is not necessarily associated with clinical psychopathology (Moulds et al., 2007).

According to Mowrer and Miller’s two-factor theory (Miller, 1951), avoidance develops through classical and operant conditioning. Individuals generalize past failures in coping to new situations, reinforcing negative cognitive and emotional patterns that perpetuate avoidance (Varangot-Reille et al., 2024). From an evolutionary standpoint, avoidance is considered a self-protective mechanism (Servatius, 2016). In the short term, it may serve as an adaptive coping strategy. For example, it has been stated that distancing from reexperiencing acute psychological strain in immersion facilitates working through it adaptively (Kross & Ayduk, 2017). However, just suppressing or putting aside those experiences often exacerbates rather than resolves underlying issues over time. Within military training or deployment contexts, delaying problem resolution can result in significant negative consequences (Edmunds et al., 2022).

During the avoidance process, individuals apply strategies that can be attributed to cognitive, emotional, and behavioral levels (Hayes et al., 1996; Wiebe, 2013). *Cognitive avoidance* manifests itself in excessive rumination or worrying, the suppression of aversive thoughts and emotions, or distraction from these thoughts or emotions (Sagui-Henson, 2017). Five cognitive avoidance strategies are described: (1) distraction by concentrating on more pleasant alternative thoughts, (2) excessive concern focused on negative thoughts about the challenge, and (3) self-punishing thoughts in the sense of self-deprecation. Furthermore, (4) social distraction(i.e., communication with close others) and (5) the reevaluation of one’s negative thoughts are stated, although adaptive on the short term (Knouse et al., 2023). Psychological evasion strategies such as rumination and worry also play a role in cognitive avoidance (Dickson et al., 2012). Both constructs are indeed associated with excessive thinking about a past or anticipated situation (Eisma et al., 2020). However, they are both classed as avoidance strategies because the individual only addresses the arising challenge and potential solutions in a nonspecific (i.e., passive), vague or abstract manner. At the same time, inactivity can be cognitively justified where an individual seeks to blame their failure on other individuals (Kaufmann et al., 2022) or groups (Beyer et al., 2017). Although it is possible to evade the challenge in the short term, this behavior rather contributes to the chronicity, or even reinforcement, of this perception in the long term. As a result of negative cognition, such as uncertainty and self-doubt, as well as impaired problem-solving abilities, rumination intensifies over time and promotes negative affect (Kim & Newman, 2023).

*Emotional avoidance* primarily entails evading associated positive or negative emotional states (Bock et al., 2024; Buhk et al., 2020). Social concerns about showing emotion or generally negative beliefs regarding emotion also play a role in this connection (Taylor et al., 2004; Tull et al., 2011). Shame and guilt, for example, are often avoided as aversive social emotions, with shame manifesting itself as an unpleasant, self-centered emotion that occurs after failure or misconduct, while guilt is associated with a negative evaluation of one’s misconduct (Cavalera & Pepe, 2014; Teroni & Deonna, 2008). Avoiding negative cognitive and emotional states often triggers negatively intensified learning processes that strengthen maladaptive coping in stressful situations (Buhk et al., 2020). Behavioral avoidance strategies can arise as a result of cognitive and emotional avoidance (Dickson et al., 2012).

*Behavioral avoidance* essentially manifests itself in social isolation, i.e., the evasion of activities and opportunities to interact with one’s social environment and is often part of avoidance-based coping strategies in which stress-induced situations are avoided through escape (Corr, 2013). Inactivity in the face of a challenge is often considered less aversive than adaptive coping attempts which are regarded as bearing the risk of failure due to limited self-efficacy (Rahimi et al., 2023).

Coping strategies based on avoidance often fail to address underlying problems or promote meaningful improvement. As a result, persistent avoidance can hinder personal development and pose risks to health, particularly in the context of stress management (Steingräber et al., 2023). In resilience training, avoiding challenges may lead to competence deficits, performance issues, and long-term stagnation (de Terte & Stephens, 2014), thereby undermining the training’s effectiveness. Consequently, reducing avoidance should be a core component of resilience-building programs (Steingräber et al., 2023). Notably, individuals are not always consciously aware of their own avoidance behaviors (Crane, Boga, et al., 2019).

In order to address this problem effectively, it is crucial to examine avoidance within the military police community undergoing training. Military police personnel play a significant role in promoting resilience within the Bundeswehr, as their specialized tasks often expose them to particularly high demands and additional risks, where errors resulting from overload can quickly lead to serious consequences. Therefore, a high level of resilience is especially crucial. During training, appropriate foundations – such as through resilience training – are intended to be established to support this goal (R. J. Gorzka et al., 2023).

Avoidance has been identified as a moderating factor in strengthening the resilience of operational forces (Bartone et al., 2017; Falon et al., 2022), with most studies addressing its role in stress management (Anderson et al., 2022). In some cases, avoidance can offer temporary relief from deployment-related stress (Zueger et al., 2022). Based on a qualitative written survey, Crane, Rapport, et al. (2019) found that Australian military police developed more effective coping strategies through *self-reflection resilience training* (SRRT), though avoidance strategies were not explicitly assessed. This may be due to their unconscious nature or social desirability bias. Nonetheless, the authors highlighted the need to examine avoidance as part of operational coping to (1) modify maladaptive mechanisms, (2) enhance resilience, and (3) foster self-reflection. With the present study, we aim to contribute to this effort. We consider it essential to further investigate avoidance within the military (police) community, intending to increase awareness of such behavioral patterns among its personnel and supporting their mitigation (Steingräber et al., 2023). A qualitative exploration of avoidance may foster a deeper understanding of the concept, enhance awareness of its implications, and help to consolidate theoretical frameworks for resilience training. Furthermore, such research can inform the development of more individualized training interventions. In addition, examining avoidance qualitatively may yield valuable insights into factors that influence readiness for change within this group (Adler et al., 2024).

To guide our research, we oriented on two key questions: (1) *How does avoidance manifest itself in the military police personnel interviewed?* and (2) *How does military police personnel deal with avoidance?*

## Methods

A qualitative approach using semi-structured interviews was chosen to answer the research questions. In preparation for the interviews, the guiding questions outlined above were derived from the central research questions, with the aim of addressing the *what*, *when*, *how*, and *why* of avoidance, as well as how the participating military police officers respond to it. To support the interviewer during the data collection process, an interview guide was developed, containing additional detailed questions and prompts. These were designed to be open-ended and low-threshold in order to encourage participants to share their experiences freely and narratively. Examples of such prompts included questions like, *How should I imagine avoidance in your case?* or *Tell me about a situation in which you have avoided or wanted to avoid something.*

To explore avoidance among military police personnel, the following central research questions were addressed: (1) *How does avoidance manifest itself in the military police personnel interviewed?* and (2) *How does military police personnel deal with avoidance?* In order to develop a comprehensive and in-depth understanding of the nature and purpose of avoidance, the contexts and forms in which it occurs, and the coping strategies employed by military police personnel, the following key guiding questions were formulated to support answering the primary research questions: (1) *What is avoidance from the viewpoint of the military police personnel interviewed*?; (2) *What does the military police personnel interviewed avoid*?; (3) *In what situations does the military police personnel interviewed show avoidance*?; (4) *In what ways does the military police personnel interviewed show avoidance*?; (5*) For what purpose does the military police personnel interviewed show avoidance*?; (6) *How does the military police personnel interviewed currently deal with avoidance*?; and (7) *How does the military police personnel interviewed plan to deal with avoidance*? An additional category was also considered: *Negative consequences of avoidance for the military police personnel interviewed*.

### Sample and design

The interviews with a total of *N* = 19 military police personnel (13 male and 6 female) were conducted at the Bundeswehr Military Police and Staff Duty School. The age of the military police personnel interviewed ranged from 18 to 46 years (*M* = 26.368; *SD* = 8.234; *Mdn* = 25). The group consisted of 11 enlisted personnel, one senior NCO undergoing officer training, 6 senior NCOs undergoing close protection specialist training and one line officer without specialization. The participating training groups were interviewed according to their availability as a convenience sample. Participation in the survey was voluntary, with personal interviews taking place during routine duty hours and lasting between 7 and 15 minutes. All personnel asked consented to take part in the interviews.

### Procedure

Participants were initially welcomed in a group setting and given an overview of the project before being invited to participate voluntarily. Each participant was then interviewed individually in a separate room. At the start of each interview, the procedure was explained, and informed consent was obtained. Participants were given ample time to review and complete the consent form, with any questions addressed immediately. Upon verbal confirmation, audio recording commenced.

Interviews began with participants stating their age and current occupation, followed by a series of key research questions. Follow-up prompts encouraged elaboration. Participants were first asked to define avoidance in their own words, then to describe workplace situations in which they had exhibited avoidant behavior. These real-life examples provided context for exploring the research questions. To ensure authentic responses, the concept of avoidance was not defined in advance.

At the end of each interview, participants could ask final questions or share additional thoughts before the recording was stopped. They were thanked for their participation and reminded of the contact information on the consent form. Lastly, the interviewer completed a post-interview documentation sheet to capture impressions relevant to the analysis.

### Data analysis

After transcription, the interviews were evaluated using *MAXQDA 24* (VERBI Software, 2024). The *Structuring Qualitative Content Analysis* method (Kuckartz & Radiker, 2023) was used to analyze the data. Initially, the key questions contained in the interview schedule were used as *a-priori categories* (Helfferich, 2022). (1) During the first review of the transcripts, they were checked for anomalies with appropriate comments being added. (2) The *a-priori categories* integrated in the interview schedule were used as main categories. (3) The transcripts were first coded in accordance with these main categories. Coding was carried out using expert ratings which reached consensus (Kuckartz & Radiker, 2023). (4) In an inductive approach, the main categories resulting from the consensus were then used to create several subcategories for each main category. (5) The subcategories were used to code text passages. (6) The initial, category-based analysis was performed on the basis of the main categories. (7) Finally, the findings could be presented on the basis of the resulting outcomes.

## Results

The interviews provided nuanced insights into how military police personnel perceive and respond to avoidance in various contexts.

### Central research question 1: How does avoidance manifest itself in the military police personnel interviewed?

The first key question—*What is avoidance from the viewpoint of the interviewed military police personnel*? – sought to clarify how they understand the concept and whether their perspectives reflect a shared or individual pattern, offering insights for future training. Participants were often initially uncertain about the open-ended nature of the question, appearing concerned with providing what they believed the interviewer wanted to hear. As one participant asked during an interview: *“Did that meet your expectations of what you wanted to hear?*”

Despite this initial hesitation, all participants ultimately articulated similar understandings of avoidance. Eighteen described it as the evasion of unpleasant tasks or situations, primarily in the sense of avoiding duties or necessary actions and preventing or delaying confrontation with aversive stimuli. According to the participants, such stimuli could be external, such as social interactions, or internal, such as revealing personal shortcomings or suppressing distressing memories and emotions. One participant stated: “Yes, contacts, maintaining contacts, or addressing certain topics with comrades, avoiding this. Avoiding contact with a comrade.” In contrast, five participants defined avoidance as a strategy for minimizing unnecessary strain. In this view, the goal was to fulfill orders and expectations as efficiently as possible to avoid pressure from superiors, including seeking less burdensome ways to meet these demands. As one participant described it: “Not doing something you should not do.”

Additionally, two participants described prioritization as a distinct concept, emphasizing that not every delay in task completion constitutes avoidance. In some cases, tasks must be postponed due to the greater urgency of other responsibilities.

Key Question 2 (What does the military police personnel interviewed *avoid?*) aimed to identify the characteristics of commonly avoided situations and activities. Participants described both external and internal avoidance triggers. Externally, ten participants referred to social situations, including interpersonal interactions with relatives during deployment or with peers in operational contexts, which two participants attributed to a lack of social skills. Five participants highlighted conflict, especially within training groups or with superiors, as a central avoidance factor, particularly when corrective action was required. Similarly, five participants reported avoiding situations involving potential criticism or negative evaluation, such as performance assessments or disciplinary conversations. Internally, emotional triggers were prominent. Eight participants reported avoiding situations due to feelings of fear or insecurity, particularly when anticipating judgment. Four participants cited fear of failure in unfamiliar training tasks as a specific avoidance motivator. One participant expressed it as follows:

If you’ve had a bad experience, then I would avoid it. And if you just don’t feel comfortable with the situation. If I’m in a situation that I’m not yet familiar with, then I’d rather avoid it for the time being.

Shame and guilt were also noted by four participants, particularly when admitting mistakes or addressing family concerns during extended absences. Ten participants described general aversion toward physically or cognitively demanding tasks. Frequently avoided activities included intense physical training, routine cleaning, equipment preparation, and official communication (each mentioned by one participant).

Key Question 3 (In what situations does the military police personnel interviewed show avoidance?) examined the situational contexts in which avoidance arises among military police personnel. Most participants described short-term, task-specific scenarios rather than ongoing conditions. Six participants reported avoidance in situations where personal errors were likely, such as during assessments or unfamiliar training tasks: “(…) then I just don’t give myself the nerve, and don’t say that you don’t know or give a wrong answer, you just say nothing.” The pressure to perform flawlessly heightened uncertainty and hesitancy. One participant described difficulty acknowledging past mistakes, citing shame as a key avoidance factor. Three participants reported avoidance in potential conflict situations – either interpersonal disputes or challenges enforcing discipline when peers failed to comply. Another three described avoiding tasks that required significant cognitive or physical effort, with anticipated strain reducing motivation and thoroughness. In contrast, six participants stated that avoidance was largely unfeasible due to constant supervision during training. However, one participant characterized avoidance as a daily strategy, particularly during transitional periods such as breaks or shift changes following demanding tasks. Finally, two participants referenced deployment settings as contexts for avoidance, either by disengaging from roommates or reducing contact with family.

Key Question 4 (*In what ways does the military police personnel interviewed show avoidance*?) explored how participants manage avoidance on cognitive, emotional, and behavioral levels. The responses revealed diverse, often overlapping strategies. Cognitively, four participants reported attributing responsibility for failures to others, either directly or through group-based diffusion. As one participant stated: “You can see that this kind of avoidance tactic comes up in your own life. You avoid admitting that you made a mistake. It’s a classic: it wasn’t me; I didn’t do it; it was the other person.” Two used downward comparisons, including imagined counterparts, to rationalize their own actions. Emotionally, nine participants described attempts to suppress or ignore stress-related feelings such as sadness or anger. When suppression failed, some experienced emotional outbursts (e.g., anger or panic), or recurring cognitive patterns like excessive worry, rumination, or catastrophizing. One participant reported the following:

At some point, it really does come out because I can’t always keep everything inside and suppress it, but at some point, there’s a point where everything just comes out and then it doesn’t end well. So that’s either a tantrum or something similar.

Behaviorally, participants used both active and passive avoidance. Three reported engaging in pleasurable distractions such as sleeping, eating, reading, or gaming. As one participant described it:

(…) I’ve been on the road all week, I know I should actually be doing this now, but then I say to myself: “I’m going to lie down or do this and that, or I’m going to eat…” And of course I put off the unpleasant things.

Seven others described turning to generally productive activities – like cleaning or exercising – as a way to delay confronting aversive situations. Two participants intentionally restructured their schedules to bypass unwanted tasks, while one admitted to withholding information to avoid conflict or responsibility. Passive avoidance was reflected in social withdrawal or silence during disagreements. Two participants described avoiding contact with peers or family during deployment to manage irritability, and three avoided escalating tensions by remaining silent in conflicts. While six participants believed that avoidance is largely unfeasible in military contexts due to structural constraints, others (two) framed avoidance as proactive burden prevention. By adhering strictly to rules, such as wearing the correct uniform, they aimed to prevent reprimands or additional stressors: “We’re in training. I want to avoid getting reprimanded or facing any consequences. I follow orders and wear the correct uniform – simple as that.”

Key Question 5 (*For what purpose does the military police personnel interviewed show avoidance?*) addressed the benefits and advantages participants gained from using avoidance strategies. The main benefits identified included the protection of the self-concept and the creation of stress-relieving breaks. Additionally, avoidance provided opportunities to prepare for, observe, and reflect on stress-inducing situations, such as practical training sessions. Four participants described avoidance as a self-defense mechanism. By avoiding social contact, they could maintain distance and prevent potential conflicts. Additionally, they could protect their self-concept by avoiding admitting mistakes, using external attribution, or postponing difficult conversations to avoid feelings of shame and guilt. One participant said: “It’s a bit of self-entitlement that you try to maintain somehow, you don’t want to admit to yourself that you’ve now made the mistake: “You don’t make a mistake like that (…).” Five participants also used avoidance to maintain a positive public image and protect their reputation within the group. This included avoiding knowledge tests to hide competence gaps or refraining from intervening in group conflicts to avoid standing out negatively. Furthermore, five participants described using avoidance to create immediate breaks from stress. Tasks they found unpleasant were either not addressed or postponed to avoid dealing with them. Examples include avoiding conflicts through restraint or waiting until the last possible moment to deliver presentations. Three participants also reported avoiding unnecessary stress through diligent task fulfillment. Four participants stated they avoided confronting stressors initially to mentally or practically prepare for the challenges ahead. For instance, in training situations involving unfamiliar tasks like house searches, they would refrain from volunteering first, choosing instead to observe others and reduce their own uncertainty when it was their turn. Finally, one participant noted that awareness of their own avoidance allowed for reflection and self-awareness, which may help address the behavior in the future.

### Central research question 2: How does military police personnel deal with avoidance?

In response to key question 6 (*How does the military police personnel interviewed currently deal with avoidance?*), fifteen participants stated that they confronted challenges directly. This was either done by addressing and discussing conflict topics head-on or by exposing themselves to uncomfortable situations, which helped alleviate stress in a short time. One participant stated:

(…) sometimes I did it in such a way that I did it first, or so that I went into the situation first and then perhaps didn’t even have time to build up this tension and nervousness. And to think so much about it.

Additionally, they mentioned preparing for training sessions to avoid active participation due to uncertainty (two participants). A structured approach was emphasized. Motivation, according to participants, was also driven by upward and downward comparisons with others, particularly in sports activities (two participants). Furthermore, participants reported withdrawing after stressful situations to reflect quietly on what had occurred (two participants). Nine participants noted that they now engage in conflict resolution sporadically and tolerate avoidance in trivial matters. Three participants described a conscious decision to avoid or accept avoidance, noting that cognitive barriers prevented overcoming such behavior. Avoidance as a means of reducing unnecessary stress was seen as a viable strategy.

Key question 7 (*How does the military police personnel interviewed plan to deal with avoidance?*) addressed participants’ future approaches to dealing with avoidance. Again, direct confrontation and engagement (socially desirable behaviors) were mentioned. As one participant stated:

But with the important stuff, I know that in the end I’ll have a better life if I can somehow manage to be more disciplined and just get through the stuff, (…). That’s why I don’t like it when I do it, but I do it anyway. But I’m aware that I’m doing it. But I try not to do it for the important things. Which doesn’t always work. I work on it.

Two participants indicated they would reinforce this behavior through social support and pressure, for example, by seeking assistance when working at the computer. There was also hope that military training would improve their discipline and stress management (four participants). Personal strategies were less defined but occasionally specified, such as the intention to improve time management using a whiteboard. One participant stated, “I would like to change something. I want to step out of my comfort zone and take on different tasks.”

An additional category (*Negative consequences of avoidance for the military police personnel interviewed*) was introduced to explore the long-term negative outcomes of the avoidance strategies described by participants. These included persistent cognitive strain, stagnation in personal development, continued avoidance behavior, dissatisfaction, and increased time pressure. Cognitively, three participants reported sustained mental overload, often driven by recurring thoughts that failed to resolve the avoided issue. Secondary strategies, such as excessive worry or rumination, were prevalent. Two participants expressed regret over missed opportunities resulting from avoidance, while one described the burden of having to mentally retain postponed tasks to prevent forgetting them. Nine participants addressed the stagnation of personal growth and the reinforcement of avoidance cycles. One participant stated: “The downside, I think, is personal growth. Because I avoid situations that would give me the opportunity to grow”. For instance, three participants associated reluctance to engage in necessary group conflicts with weakened group cohesion. One reported emotional outbursts caused by suppressed personal needs, and another described how avoidance led to further avoidance, inhibiting adaptive coping. Five participants noted persistent dissatisfaction, linked to a discrepancy between their ideal self-concept, such as being task-oriented, and their actual behavior. One participant highlighted how unresolved tasks, like delayed phone calls, triggered recurring guilt. Finally, time pressure was identified by three participants as a negative outcome. Procrastination led to increased stress and impaired task execution. Two participants reported that under time constraints, critical items were forgotten during equipment preparation.

In summary, a detailed picture of avoidance was obtained from the interviews with military police personnel. It became clear that both in the professional and the private context, the respondents avoided inner and outer aversive states associated with challenges. They act in both active and passive ways at the cognitive, emotional, and behavioral levels. The military police personnel interviewed made it clear that they were aware of the advantages and disadvantages of avoidance strategies. The interviews revealed evidence of initial engagement with self-reflection but also continued avoidance. Some of the respondents stated that they hoped for support in countering avoidance to further their personal development.

## Discussion

This study aimed to examine and structure the use of avoidance strategies by military police personnel in the context of resilience research and use it as a basis for contributing to theory formation and to deriving practical implications for resilience enhancement. To this end, a qualitative survey in the form of semi-structured interviews was carried out.

The findings highlight the complexity of avoidance among military police personnel, particularly regarding the balance between short-term coping and long-term effectiveness. Participants distinguished between functional prioritization – where urgent tasks are addressed before less critical ones – and true avoidance, which involves the deliberate postponement or circumvention of aversive experiences (Schmid et al., 2015; Waugh et al., 2020). While short-term avoidance may offer temporary relief in high-pressure contexts, its prolonged use risks negative psychological and operational consequences (Chao, 2011).

Avoidance was often triggered by anticipated failure, unknown challenges, or emotionally aversive states such as shame or guilt (Cavalera & Pepe, 2014; Teroni & Deonna, 2008). In the dynamic and hierarchical environment of military service, such triggers are recurrent, necessitating continual psychological adaptation (Hill et al., 2024). Several participants reported avoiding conflictual or evaluative situations to preserve their self-concept or social standing, especially in group settings. Yet, this avoidance undermines opportunities for constructive feedback and hinders personal development (Frese & Keith, 2015). Similarly, unresolved interpersonal tensions were cited as barriers to group cohesion and resilience, consistent with prior findings on the social costs of conflict avoidance (Dijkstra et al., 2009; Johnson et al., 2001).

Cognitive and behavioral avoidance strategies were diverse. Some individuals engaged in distraction, suppression, or rumination – mechanisms that offer short-term relief but contribute to long-term cognitive strain and emotional dysregulation (Katsumi & Dolcos, 2020; Stanisławski, 2019). Others employed disengagement strategies such as deflecting responsibility or procrastination (Beyer et al., 2017; Kaufmann et al., 2022; Rahimi et al., 2023). Passive patterns like social withdrawal or concealment of needs further reflected a perceived lack of agency in managing demands. These strategies align with the literature on maladaptive coping, where avoidance leads to persistent stress and impaired adaptation (Chao, 2011; Kim & Newman, 2023).

Nevertheless, avoidance was also framed by participants as a means of short-term self-regulation. Respondents reported using it to protect their self-image, create moments of rest, and allow time for preparation. While such breaks can be beneficial if used adaptively, postponement without resolution tends to perpetuate stress (Siregar, 2023). Encouragingly, some participants demonstrated reflective awareness of their avoidance, an important precursor to behavioral change (Greif, 2008).

Although direct responses often downplayed the presence of avoidance, descriptions of situational behavior and emotional regulation revealed indirect indications of avoidance, particularly in ambiguous or high-stakes contexts. When addressing avoidance, participants reported three primary patterns: active confrontation, ambivalent intentions, and continued avoidance. Adaptive confrontation involved directly facing challenges and using temporary distancing for processing, a practice associated with emotional regulation and long-term learning (Cova et al., 2019; Kross & Ayduk, 2017). However, many responses indicated vague intentions for future improvement without concrete plans, suggesting that while awareness exists, implementation remains limited. Finally, the cumulative effects of avoidance were acknowledged by the participants themselves. Persistent cognitive load, stagnation in personal development, dissatisfaction, and mounting time pressure were common consequences. These findings align with broader research on the long-term costs of avoidance for performance, well-being, and resilience capacity (Jain et al., 2015; Nindl et al., 2018; Yeager & Dweck, 2012).

### Limitations

The preliminary assumptions were indeed developed on a scientific basis. Ultimately, however, they limited the number of new insights that could be obtained because they were already integrated in the interview schedule. As a result, the interviews took a different course, depending on the responses. For example, from a didactic-methodical perspective, avoidance-affirmative attitudes borne out of a positive definition of avoidance (*avoidance of unnecessary stress)* posed an obstacle to obtaining information. Concerning avoidance, situational contexts were mostly taken into account in the analysis. Consideration of the respondents’ personality or socialization as military police personnel would also have been a valuable aspect (Carver & Connor-Smith, 2010; Connor-Smith & Flachsbart, 2007).

Overall, participants reported few clear instances of avoidance. While the study focused on avoidance, this does not necessarily indicate it is the primary threat to resilience. Still, its presence, however limited, suggests it remains a relevant factor worth addressing in training and future research.

The discrepancy between ranks may also be a problem, considering that the interviews were conducted by an officer in uniform (Staal, 2023). In the Bundeswehr, there is invariably a certain reticence in communication with higher-ranking military personnel. This might also have influenced the willingness to provide information and the comfort of the participants. The relatively infrequent direct acknowledgment of avoidance may also reflect normative pressures within military culture that discourage the disclosure of perceived weakness or failure to act. As Crane, Boga, et al. (2019) suggest, avoidance is not always consciously recognized or may be underreported due to social desirability biases.

### Implications

It can be assumed that avoidance affects military police resilience training. While the long-term use of avoidance strategies may undermine resilience, it is important to recognize that, in certain operational contexts, avoidance can serve a functional role. Specifically, during high-intensity situations that demand peak performance, such as live operations or crisis management, temporary avoidance may help personnel compartmentalize emotional responses and maintain focus. While focusing on reducing long-term avoidance, military police resilience training should offer a dual perception of avoidance including adaptive short-term stress management techniques for time-pressured, high-stakes contexts (Steingräber et al., 2023).

Hence, it is important to offer military police personnel undergoing training psychoeducation on avoidance in the context of a dynamic view of resilience. While all individual definitions of avoidance can and should be given their due place, the negative framing of *avoidance* should be clearly illustrated at the end of this training phase. In addition, there should be reflection on the advantages and disadvantages of avoidance. Awareness of one’s avoidance and its consequences can serve as the starting point for resilience training, possibly in the form of self-authoring, i.e., reflection on specific goals in keeping with one’s internal frame of reference and on skills and competencies to be developed (Cavallaro & French, 2021). This process is influenced by motivational and volitional mechanisms (Falon et al., 2021). Moreover, it is useful to consider the characteristics of situations and tasks that trigger inner aversive states in the participants. Particular attention must be paid to the context of such settings, especially in case of severe and long-term stress, such as during missions abroad (Zimmermann et al., 2014). With the help of previously completed training courses or personal experiences, cognitive, emotional and behavioral avoidance strategies can then be examined to develop individual counter strategies. In this context, it is essential that the military personnel actively cooperate, while particular attention must be paid to the fact that the way avoidance manifests itself is individual in nature, as there are different ways of controlling it individually (Vanhove et al., 2016).

Setting specific objectives allows for the application of motivational and volitional mechanisms to counteract avoidance, thereby supporting participants’ perceived self-efficacy (Kreibich et al., 2022). To establish a universal goal, however, the question must be addressed as to what level of avoidance is acceptable, acknowledging that it may never be fully eliminated. Simultaneously, implementing social support measures to strengthen team cohesion should be prioritized, as enhanced interpersonal dynamics and collective resilience may reduce avoidance at the group level (Chapman et al., 2021). Moreover, any intervention should assess whether individual military police personnel are both willing and able to commit to genuine behavioral change. If such readiness is lacking, tailored individual support may be necessary (Adler et al., 2024). Further, future research should also explore how avoidance manifests in other military contexts beyond the military police. Comparative studies could illuminate whether and how military police differ from other military personnel in their coping strategies and exposure to stressors.

## Data Availability

The data supporting the findings of this study consists of interviews with active-duty military police personnel. Due to legal restrictions and the sensitive nature of the data, they cannot be made publicly available. Access to this data is restricted to ensure the privacy and confidentiality of the participants, in compliance with relevant legal and ethical guidelines.
